# Peter Tontonoz honored with the 2022 ASCI/Stanley J. Korsmeyer Award

**DOI:** 10.1172/JCI159675

**Published:** 2022-04-01

**Authors:** Elyse Dankoski

The American Society for Clinical Investigation (ASCI) has selected Peter Tontonoz to receive 2022’s Stanley J. Korsmeyer Award, recognizing his foundational discoveries in lipid metabolism and his dedication to excellence in mentorship (Figure 1). Dr. Tontonoz is the Frances and Albert Piansky Endowed Chair and Distinguished Professor of Pathology and Laboratory Medicine and of Biological Chemistry at the University of California Los Angeles (UCLA), Co-Director of the UCSD/UCLA Diabetes Research Center, and Vice Chair for Research in UCLA’s Department of Pathology and Laboratory Medicine. His research has revealed that the lipid-activated nuclear receptors LXR (liver X receptor) and PPAR (peroxisome proliferator–activated receptor) coordinate lipid metabolism and modulate immunity and inflammation, demonstrating important roles for LXR and PPAR signaling pathways in atherosclerosis, insulin resistance, and immune tolerance. Recently, Dr. Tontonoz spoke with the *JCI* about his extraordinary scientific career.

*JCI*: What first sparked your interest in science, and how did you eventually decide to pursue an MD/PhD?

Tontonoz: I remember taking a high school science course where I had to design experiments to show that paramecia would chemotax. I enjoyed that sort of practical experimentation, and that was my first insight into what actually doing science is like. When I got to college, I worked on yeast protein kinases for an assistant professor who had just been hired at Wesleyan. From that first moment setting foot in a molecular biology laboratory, I knew that that’s what I wanted to do. I just thought being in the lab was the greatest thing, and I was there all the time. I stayed at school over the summers so I could continue to work in the lab, and basically, I haven’t left the lab since. Even when I went to medical school, I was still always in the lab. I found the subject matter intellectually interesting, but practically, I really liked being in the laboratory and doing experiments.

I’d never heard of such a thing as an MD/PhD when I went to college. I found out about that only during college, learning more about what the options were. It seemed like a good option for someone like me who really was very interested in science and academically interested in medicine. But I always knew I would do science as some component of my life from the moment I stepped in a lab.

*JCI*: Your PhD mentor, Bruce Spiegelman, has described your PhD research as a turning point for his lab (1). Would you tell us the story of those findings?

Tontonoz: The work was interesting to me because it really was a big open question. Back then, it was the stage in which people were identifying tissue-specific transcription factors and showing that those transcription factors did important things in different tissues. A few years earlier, MyoD, the transcription factor that makes muscle, had been discovered and characterized (2). I remember reading that paper thinking, “Wow, I would love to discover this for fat,” and Bruce was thinking the same thing.

So we set out to try to identify the master transcription factor for fat using a two-pronged approach: we picked a fat cell–specific gene, *aP2*, and tried to figure out the transcription factors regulating that gene, a very laborious process. We cut up fragments of the gene one by one and ran reporter assays with the fragments of DNA hooked up to a reporter. We found this little region in the promoter that, if hooked up to any gene, would confer fat-specific expression in cultured cells. We then took a more molecular biology–based approach: the sequence looked like a nuclear hormone receptor binding site, so we went looking for members of the nuclear hormone receptor family that were expressed in fat. We immediately realized that one of them, PPARγ, was pretty much only expressed in fat, and that became the likely candidate. When we got back the results of sequencing the purified protein, the purified protein was PPARγ (3, 4).

It’s one of those nice examples of a big discovery that turned out to be right. It could have been that we identified a transcription factor that was a minor player, and something else was the master regulator... But PPARγ really is the master regulator of fat, and it’s been replicated widely. It’s still probably the biggest discovery of my career. I’m still trying to live up to that. But it was a nice start.

*JCI*: Your postdoctoral work in Ron Evans’s lab took a more translational angle to studying PPARγ and other nuclear receptors, showing that they have roles in cardiovascular and metabolic disease.

Tontonoz: There is this dogma that after your PhD, you have to do something completely different for your postdoc. You have to switch fields. You can’t stay in the same field for some reason. I always tell students, “That’s ridiculous. Do whatever you want and follow what you like.” I started out working on fat and discovered a nuclear receptor, and then I wanted to work in a lab focused on nuclear receptors.

At that point I was a pathology resident, so I was starting to ask questions that were a bit more disease and medically relevant. The idea of applying basic science knowledge about nuclear hormone receptors and PPARs to relevant disease contexts interested me. It turns out in addition to fat, PPARγ is very highly expressed in macrophages. Macrophages are very important for atherosclerosis; they take up cholesterol in the artery and become foam cells. We showed that lipid-activated nuclear receptors are very important in controlling gene expression in the macrophages in the setting of atherosclerosis and opened up the idea that these active transcription factors in macrophages could be targeted by drugs and by small molecules (5). There really hadn’t been a lot of transcription factor work done on macrophages in atherosclerotic lesions at that time. Because nuclear hormone receptors are eminently druggable, the idea that you could target them directly was very exciting. The other component of that work was identifying some oxidized lipids known to be present in the setting of atherosclerosis as activators of the nuclear hormone receptors (6).

*JCI*: We now know that PPARs are involved not only in metabolic and cardiovascular diseases but also in immunity, neurodegenerative diseases, and cancer. How do you approach your current work to connect the dots between your interest in basic science and its clinical context?

Tontonoz: What I saw with Bruce’s and Ron’s work was that doing good basic science leads to results that have clinical importance. Going the other way is harder, I think; if you don’t know what fundamental biology you’re studying, you don’t tend to make a lot of progress on the translational side. So that’s how I run my lab. I ask the basic science questions and look for how the basic biology we uncover is relevant to the clinical situation in the animal. My ideal story goes all the way from the basic molecular biology discovery to the physiological demonstration of importance for both biology and disease in the animal.

What I found to be very fun over the last 10 years or so is deorphanizing proteins. We used the LXR and the PPAR transcription factors as a roadmap to find genes that were important in cardiovascular cholesterol metabolism and fatty acid metabolism. We basically figured out all the genes that those 2 transcription factors regulate and went one by one over the years figuring out what each of those little downstream components do, and they all do something interesting. Almost every gene regulated by LXR has an interesting phenotype if you knock it out in a mouse. Based on that, we just kept knocking them all out. For some, we were able to take proteins with unknown function and figure out what they do, and that was tremendously satisfying.

The E3 ligase IDOL was a completely unknown protein, and lo and behold, we found that it controls the LDL receptor at a posttranslational level (7). People had worked on the LDL receptor since Brown and Goldstein won the Nobel Prize but this was still there to be discovered. Most recently, we discovered the Aster-A, -B, and -C proteins, which move cholesterol from the plasma membrane to the ER (8). That’s probably the biggest discovery that my own lab has made, because it’s a truly fundamental process. Again, we knew this had to exist, but nobody knew which proteins did it.

*JCI*: How did working with Bruce Spiegelman and Ron Evans influence your approach to mentorship?

Tontonoz: They’re both outstanding scientists in their own right. My mentoring style is sort of a meld between the two. I think the most important thing is to inspire and excite people about what you’re doing, and more than anything else that’s what I try to do as a starting principle. I want an environment where it’s fun to do science and people want to come to work every day, because that’s what I remember about working in Bruce’s and Ron’s labs. I wanted to be there all the time, I wanted to do the next experiments, and I was really excited about doing it. I was sure that if I worked hard enough, it was going to lead somewhere and be productive. I’m very careful about my lab environment, and that comes from my experience with Bruce and Ron. It becomes self-sustaining, because when people in the lab are happy, the lab becomes its own recruiting tool.

There’s nobody more excited about science than Bruce Spiegelman. That was infectious. I was inspired from day one, and it hasn’t stopped. I still think about: “What would Bruce do?” I always tell the example that Bruce would stare at the autoradiograph for hours. He would see the faint little band, which was the hint of what would become the *Science* paper. Bruce could see that little blob from a thousand miles away. He’d say, “I think that’s something interesting,” and he would be right. He had great instincts, and he was passionate about it, and we believed him. We were all in there looking at the autorad with him.

*JCI*: What does winning the Korsmeyer Award mean to you?

Tontonoz: Obviously, this is one of the biggest honors you can get, especially as a physician-scientist. It means a ton to me because I have been in the room selecting people for the Korsmeyer Award. I never in a million years thought that I would be in consideration for this, and there are people who I really, really look up to on the list of past recipients, so it’s very humbling. The other thing is, I love the ASCI, and I spent a long time with the organization.

If you had asked me, “What’s one recognition that would mean a lot to you, other than the Nobel Prize?” I would have mentioned the Korsmeyer Award. It’s particularly special this year because I actually nominated one of my trainees, Tamer Sallam, to the ASCI, and he’s going to be inducted this year. I’m as proud of that. It makes it doubly special to have him there in the audience.

## Figures and Tables

**Figure 1:**
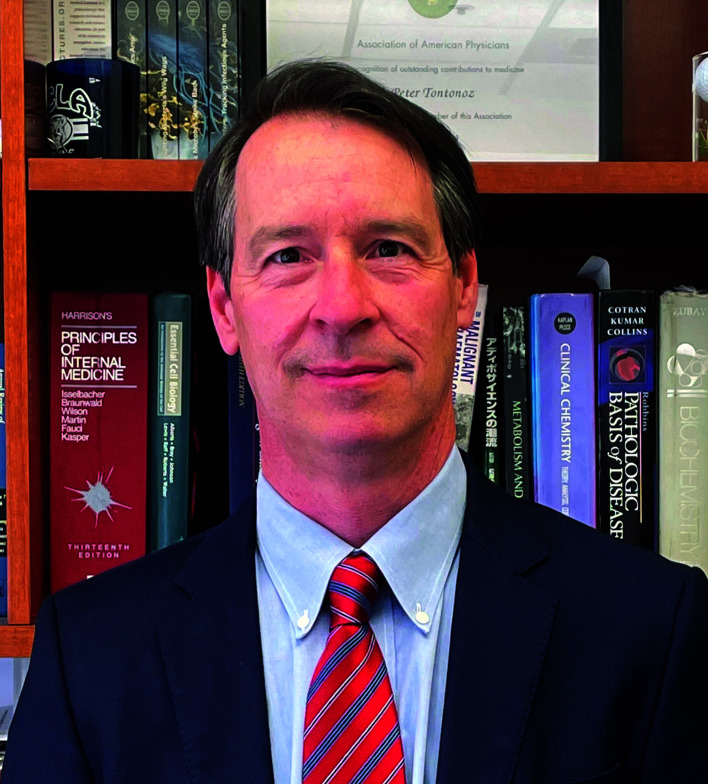
Peter Tontonoz is the recipient of the 2022 ASCI/Stanley J. Korsmeyer award.

## Version 1 04/01/2022

Electronic publication
